# Alterations of RNA-binding protein *found in neurons* in *Drosophila* neurons and glia influence synaptic transmission and lifespan

**DOI:** 10.3389/fnmol.2022.1006455

**Published:** 2022-11-11

**Authors:** Wei-Yong Lin, Chuan-Hsiu Liu, Jack Cheng, Hsin-Ping Liu

**Affiliations:** ^1^Graduate Institute of Integrated Medicine, College of Chinese Medicine, China Medical University, Taichung, Taiwan; ^2^Department of Medical Research, China Medical University Hospital, Taichung, Taiwan; ^3^School of Chinese Medicine, College of Chinese Medicine, China Medical University, Taichung, Taiwan; ^4^Graduate Institute of Acupuncture Science, College of Chinese Medicine, China Medical University, Taichung, Taiwan

**Keywords:** *fne*, RNA-binding protein, neuron, glia, synaptic plasticity

## Abstract

The *found in neurons* (*fne*), a paralog of the RNA-binding protein *ELAV* gene family in *Drosophila*, is required for post-transcriptional regulation of neuronal development and differentiation. Previous explorations into the functions of the FNE protein have been limited to neurons. The function of *fne* in *Drosophila* glia remains unclear. We induced the knockdown or overexpression of *fne* in *Drosophila* neurons and glia to determine how *fne* affects different types of behaviors, neuronal transmission and the lifespan. Our data indicate that changes in *fne* expression impair associative learning, thermal nociception, and phototransduction. Examination of synaptic transmission at presynaptic and postsynaptic terminals of the larval neuromuscular junction (NMJ) revealed that loss of *fne* in motor neurons and glia significantly decreased excitatory junction currents (EJCs) and quantal content, while flies with glial *fne* knockdown facilitated short-term synaptic plasticity. In muscle cells, overexpression of *fne* reduced both EJC and quantal content and increased short-term synaptic facilitation. In both genders, the lifespan could be extended by the knockdown of *fne* in neurons and glia; the overexpression of *fne* shortened the lifespan. Our results demonstrate that disturbances of *fne* in neurons and glia influence the function of the *Drosophila* nervous system. Further explorations into the physiological and molecular mechanisms underlying neuronal and glial *fne* and elucidation of how *fne* affects neuronal activity may clarify certain brain functions.

## Introduction

The embryonic lethal abnormal visual system-like (ELAVL/Hu) proteins are a family of RNA-binding proteins (RBPs) with three highly conserved RNA recognition motifs (RRMs) that mediate the post-transcriptional regulation of gene expression through alternative RNA splicing, localization, stability, translation, and metabolism (Colombrita et al., 2013). Humans possess four ELAVL/Hu protein-encoding genes; specifically, a ubiquitous member (*ELAVL1/HuR*) and three predominantly neuronal members (*ELAVL2/HuB*, *ELAVL3/HuC*, and *ELAVL4/HuD*), which are often used as reliable biomarkers for neurons. The *Drosophila* ELAV family comprises three members: *elav*, *RNA-binding protein 9* (*Rbp9*), and *found in neurons* (*fne*). These proteins are highly enriched in neurons, and RBP9 is also found in gonads (Colombrita et al., 2013). The ELAV protein is encoded by *elav*, and deletion of *elav* leads to defects in the visual system and to embryonic lethality (Jimenez and Campos-Ortega, 1987). ELAV is mostly found in the neuronal nucleus and is involved in the stability of mRNA, alternative splicing, and in the cleavage of the polyadenylation site of the 3’ untranslated region (3’-UTR) (Koushika et al., 1996; Carrasco et al., 2020). The RBP9 protein encoded by *Rbp9* is present not only in neuronal nuclei but also in the cytoplasm of cystocytes during oogenesis for the necessity of germ cell differentiation (Kim-Ha et al., 1999). No gross neural developmental defects have been observed in *Rbp9* null mutation, but reduced locomotor activity, shorter lifespan, and defects in the blood-brain barrier and female sterility have been observed (Kim-Ha et al., 1999; Kim et al., 2010).

The third *elav*-related gene in *Drosophila*, *fne*, is present throughout development but after *elav* expression. The FNE protein is cytoplasmic and is restricted to neurons of the central and peripheral nervous systems (PNS) (Samson and Chalvet, 2003). The functional necessity of ELAV and FNE proteins to performing neural 3’-UTR alternative polyadenylation (APA) has been demonstrated, and in the absence of *elav*, FNE can rescue ELAV-mediated APA (Carrasco et al., 2020; Wei et al., 2020). Null mutants of *fne* were viable in adults: morphological defects were not apparent, but overexpression of *fne* in neurons resulted in an extreme decrease in the viability and phenotypic defects in the wings and legs (Samson, 2008). *fne* is also involved in the male courtship performance and the development of sex-specific nervous systems, and is required to restrict axonal extension of the beta lobes in adult mushroom body, a brain structure essential for the learning and memory (Zanini et al., 2012; Sun et al., 2015).

The physiological function of glia in *Drosophila* has been documented (Yildirim et al., 2019). In the central nervous system (CNS), similar with mammalian glia interacting with neurons, *Drosophila* glia are required for the regulation of neuronal development and differentiation in the brain and in the visual system (Edwards and Meinertzhagen, 2010). Glial cells also provide various supportive functions to neurons, including the modulation of electrical transmission or ion homeostasis, metabolic support of neurons, BBB formation, and responses to neuronal injury or infection (Logan, 2017; Nagai et al., 2021). In the PNS, the peripheral glia play an important role in coordinating synaptogenesis and shaping the synaptic connection in the neuromuscular junction (NMJ) by releasing glia-derived factors (Ou et al., 2014).

Studies on *fne* have mainly focused on neurons. However, the function of *fne* in glia has not yet been identified. In the present study, we investigated the physiological and molecular effects of *fne* in both neurons and glia, and our results revealed that changes in *fne* expression impaired phototransduction, learning performance, and thermal nociception. Changes in *fne* expression also affected the synaptic transmission and plasticity of the larval NMJ and preferentially acted at the presynaptic terminal, while knockdown of *fne* in the glia was highly influential. Our results suggest that expression of *fne* in the glia is essential for neurotransmission and elucidate the mechanism of *fne* in flies, possibly by extension, in mammals.

## Materials and methods

### Fly culture and strains

*Drosophila* strains were raised on regular cornmeal food under a 12-hr light/dark cycle with consistent 50–60% humidity at 25°C unless otherwise noted. The wild-type strain was *w*^1118^. The fly strains used in this study, including *GMR-GAL4* (photoreceptor-specific, stock #1104), *tub-Gal80*^ts^** (stock #7018 and 7019), *TrpA1*^1^ (stock #26504), and *UAS-fne* (stock #6897) (Chalvet and Samson, 2002) were obtained from the Bloomington *Drosophila* Stock Center (BDSC), and *fne-*knockdown strain (*UAS-fne-IR*, stock #101508) (Alizzi et al., 2020) was obtained from the Vienna *Drosophila* Resource Center (VDRC). The strains, *elav^*C*155^-GAL4* (neuron-specific), *C57-GAL4* (muscle cell-specific), and *repo^7415^-GAL4* (glia-specific), which were outcrossed with *w*^1118^ (*isoCJ1*) for at least five generations, were provided by Dr. Hui-Fu Guo. Functioning of *UAS-fne* and *UAS-fne-IR* lines was verified in Supplementary Figure 1.

Because overexpressing *fne* under *repo-GAL4* and *C57-GAL4* drivers results in larval/pupal lethality, we used the TARGET expression system to temporally control *fne* expression under the specific *GAL4* drivers combined with temperature-sensitive *GAL80*^ts^** (*tub-GAL80*^ts^**) (McGuire et al., 2004). The flies were reared at the permissive temperature (18°C) during the developmental stages and shifted to the non-permissive temperature (25 or 29°C) after eclosion to initiate *GAL4* transcription of *UAS* transgenes.

### Electroretinogram analysis

The Electroretinogram (ERG) test was performed as previously described (Cosens and Manning, 1969). Briefly, each fly was captured in a pipette tip. A reference electrode was contacted with the head and a recording electrode was attached to the fly’s compound eye. The fly eye was exposed to a 1.5 W white-light LED which is programmed to a repetitive on-off cycle of 2–6 s. Signals were recorded by Axon Instruments GeneClamp 500 Voltage Patch Clamp Amplifier (Molecular Devices, USA) and analyzed by Axon Instruments pCLAMP software (Molecular Devices, USA). The ERG was recorded from males which were reared at 18°C and shifted to 29°C after eclosion for 5 days to induce *fne* and *fne-IR* expression in photoreceptors and glia under *GMR-GAL4* and *repo-GAL4* combined with *tub-GAL80*^ts^** (referred as *GMR^ts^* and *repo^ts^*), respectively. Data were collected and analyzed at least 6 on-off cycles per fly.

### Olfactory associative learning assay

The flies with *fne* and *fne-IR* were reared at 18°C and after eclosion, shifted to 25 or 29°C to induce transgenes expression in neurons and glia under *elav^ts^* and *repo^ts^*, respectively. A single training trial was performed according to previous studies (Dubnau et al., 2001). Briefly, approximately 100 flies were exposed sequentially to two odors, either 3-octanol (OCT, Sigma-Aldrich) or 4-methylcyclohexanol (MCH, Sigma-Aldrich). Flies exposed to the first odor (conditioned stimulus, CS +) were paired with 80 V electric shocks and then received a second odor (CS-) without shocks. Learning ability was determined immediately after training. To perform the test, the trained flies were trapped into the choice point of a T-maze in which they were exposed simultaneously to OCT and MCH. A performance index (PI) was calculated to represent the conditioned odor avoidance. The PI = (# of CS- – # of CS +)/(# of total flies), and the final PI was calculated by averaging the PI in which OCT was the shock-paired odor and one in which MCH was the shock-paired odor. A PI of zero (no learning) indicated a 50:50 distribution, and a PI of 100 showed a 0:100 distribution away from the CS + odor.

### Nociceptive test

For the avoidance of noxious heat, we used the behavior paradigm as previously described (Neely et al., 2011). Briefly, approximately 20 male flies were placed in a sealed peri dish chamber. The chamber was then floated on a 46°C water bath for 1 min 30 s. The bottom of the chamber was heated to 46°C, whereas the top of chamber was measured to be 31°C. All tests were performed under red light. Flies that move away from the 46°C bottom surface were considered to be capable of avoidance of heat challenge. The percentage of avoidance in response to noxious temperature was calculated by counting the number of flies that avoid the noxious temperature compared to the total number of flies in the chamber. The flies with *fne* and *fne-IR* were maintained at 18°C and the newly *eclosed* adult males were shifted to 29°C for 5 days and 25°C for 10 days to induce transgene expression in neurons and glia under *elav^ts^* and *repo^ts^* control, respectively.

### Electrophysiology of the larval neuromuscular junction

Electrophysiological recordings of two-electrode voltage clamp were performed as previously described (Guo and Zhong, 2006). Wall climbing third-instar larvae were chosen for dissection at 25°C in Ca^2+^-free hemolymph-like (HL-3) solution containing the following (in mM): 70 NaCl, 5 KCl, 4 MgCl_2_, 10 NaHCO_3_, 5 trehalose, 5 HEPES, and 115 sucrose. For recordings, HL-3 solution was supplemented with 0.2 mM CaCl_2_. All recordings were made at the longitudinal muscle fiber 12 (M12) of segments A3-A5. To elicit evoked excitatory junction currents (EJCs), the segmental nerve was stimulated with a suction electrode at 1.5 times of the stimulus voltage required for a threshold response. Continuous recordings were made to measure the EJCs and miniature EJCs (mEJCs) while the nerve was stimulated at the baseline frequency of 0.05 Hz. For induction of short-term facilitation (STF), short trains of 20 repetitive stimulation were delivered at the frequency of 0.5–25 Hz. Current signals were amplified with an Axoclamp 2A amplifier (Molecular Devices, USA), filtered at 0.1 kHz on-line, and converted to a digital signal using a Digidata 1320A interface (Molecular Devices, USA) and pCLAMP software (Molecular Devices, USA). Stimulation of nerves was achieved by a Grass Instruments S88 Stimulator (Grass Instruments, USA). EJCs were determined by averaging consecutive EJCs in 0.05 Hz during the 5 min recording period, while mEJCs were analyzed from continuous recordings of 1 min. Values of average mEJC amplitudes were determined and quantal content is calculated as dividing average EJC amplitude by average mEJC amplitude. For STF, the EJC amplitudes of the last 10 responses in each train were averaged and normalized to the average EJC amplitudes at 0.5 Hz. The experiments were performed at 25°C. For overexpressing *fne* under *C57*^ts^** and *repo^ts^*, the flies were reared at 18°C started from embryonic stages, shifted to 29°C for 1 day, and performed electrophysiology.

### Longevity assay

The flies were expressed *fne* and *fne-IR* under *elav^ts^* and *repo^ts^* drivers. All flies were maintained at 18°C and shifted to 25°C after eclosion. Emerging adult flies were collected and separated by sex. Around thirty to thirty-five flies were kept in a vial. Four repeat vials, comprising at least 120 flies in total were conducted for each genotype. Food vials were replaced every 3–4 days, and dead flies were counted at that time.

### Data analyses and statistics

Statistical analysis of associate learning and longevity assay were performed by a Student’s *t*-test and Log-rank test, respectively. For multiple comparisons of ERG, nociceptive test, and electrophysiology, data were performed using the one-way analysis of variance (ANOVA) followed by Turkey *post-hoc* test. The results represent as mean ± standard error mean (SEM) from at least three biological replicates. *p*-value less than 0.05 was considered statistical significance.

## Results

### Electroretinogram amplitudes are influenced by *fne*

**FIGURE 1:**
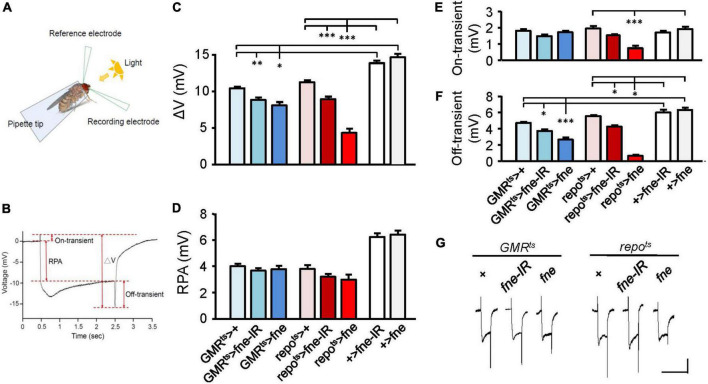
*fne* is involved in the light-induced synaptic transmission in neurons and glia. **(A)** A schematic diagram of ERG recording. **(B)** The components of a representative ERG signal contain the ΔV (the difference between on- and off-transient spikes), receptor potential amplitude (RPA), and on-transient (highest peak from baseline) and off-transient (lowest peak from baseline) spikes. Quantification of **(C)** ΔV, **(D)** RPA, **(E)** on-transient, and **(F)** off-transient spikes. **(G)** The ERG trace of each genotype. Calibration: 2 mV, 5 s. Error bars represent SEM. *n* = 10 for each group. *p < 0.05, **p < 0.01, ***p < 0.001.

Photoreceptors respond to periodic light exposure by converting light signals into electric currents. A periodic electric signal can thus be recorded by an extracellular ERG (Figure 1A). The typical ERG waveform of a wild-type fly can be divided into four components (Figure 1B). The receptor potential amplitude (RPA) reflects the depolarization of the photoreceptors during stimulation with light. The on- and off-transient spikes, which occur at the beginning and end of a flash of light, respectively, indicate the successful transmission of a signal from photoreceptors to their postsynaptic partners in the lamina (Montell, 1999). The quantity ΔV represents the amplitude between on- and off-transient spikes.

To test whether *fne* affected light-evoked responses in the retina, we used *GMR^ts^* and *repo^ts^* to temporally control *fne* and *fne-IR* expression in the photoreceptors and glia, respectively (Figures 1C–G). The photoreceptors expressing *fne* and *fne-IR* (*GMR*^ts^** > *fne* and *GMR*^ts^** > *fne-IR*) revealed slightly affected electric potential, as indicated by the smaller ΔV and off-transient amplitudes. The flies with *fne* and *fne-IR* expression in glia (*repo*^ts^** > *fne* and *repo*^ts^** > *fne-IR*) exhibited decreased ΔV, on- and off-transient amplitudes. These results indicate that *fne* affects neurotransmission in both photoreceptors and glia and that changes in *fne* expression in glia cause more severe ERG defect than in neurons.

### Overexpression of *fne* in neurons and glia impairs cognitive ability

**FIGURE 2:**
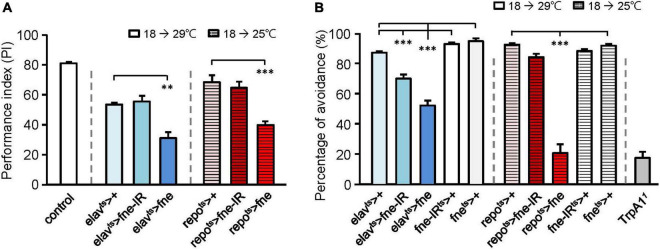
Effects of *fne* on cognitive behavior and thermal nociception. **(A)** The learning PI of *fne* and *fne-IR* expressing in neurons and glia. The control fly is 25°C 5-day-old *Canton-S* strain for experimental calibration. *n* = 5–6 for each group. **(B)** The percentages of avoidance in response to noxious temperature of *fne* and *fne-IR* expressing in neurons and glia. The *TrpA1* mutant (*TrpA1*^1^) fly is a positive control (25°C, 5-day-old). *n* = 10 for each group. Error bars represent SEM. **p < 0.01, ***p < 0.001.

Because changes in *fne* expression affect photoreceptor transmission, we attempted to determine the effects of *fne* on behavior. A single cycle of olfactory associative training was conducted to evaluate the learning performance index (PI; Figure 2A). After eclosion, the temperature was raised from 18 to 29°C for 10 days, and the flies started to overexpress (*elav*^ts^** > *fne*) or knockdown (*elav*^ts^** > *fne-IR*) *fne* in neurons. The PI of the *elav*^ts^** > *fne-IR* flies was similar to that of the control group (*elav*^ts^** > +, 55.5 ± 3.8 vs. 53.5 ± 1.1), but the *elav*^ts^** > *fne* flies exhibited a lower PI than did the control (31.1 ± 4.0, *p* < 0.01). We also evaluated learning ability when *fne* expression in glia began to change during the adult stage. Because the overexpression of *fne* in glia at 29°C caused extremely low viability, we kept the flies at a lower non-permissive temperature of 25°C for 14 days. Our data revealed that the *repo*^ts^** > *fne* flies had significantly lower PIs than did the control flies (39.8 ± 2.5 vs. 68.4 ± 4.6, *p* < 0.001), but *repo*^ts^** > *fne-IR* flies showed non-significant difference. These results indicate that the overexpression but not knockdown of *fne* both in neurons and glia impairs learning behavior.

### Changes in *fne* expression reduce thermal sensation

Having determined that changing *fne* expression impairs cognitive ability, we tested another behavior, thermal nociception. When the flies were exposed to a noxious temperature (46°C) at the bottom surface of a peri dish chamber, they rapidly moved to the top, which had a subnoxious temperature, to avoid being harmed by the heat. A low percentage of avoidance indicated that the flies were less sensitive to the noxious heat (Neely et al., 2011). When *fne* and *fne-IR* were expressed in neurons at 29°C for 5 days, the percentages of avoidance appeared lower in the *elav*^ts^** > *fne-IR* and *elav*^ts^** > *fne* groups (69.4 ± 2.7% and 51.7 ± 3.3%, respectively) than in the control groups (*elav*^ts^** > +, 86.5 ± 1.0%; *fne-IR*^ts^** > +, 92.1 ± 1.0%; *fne*^ts^** > +, 94.1 ± 1.6%; Figure 2B). After eclosion, the flies were kept at 25°C for 10 days to express *fne* in the glia, and the percentage of avoidance for the *repo*^ts^** > *fne* flies was 20.5 ± 5.8%, which were lower than the controls (*repo*^ts^** > +, 91.8 ± 0.8%; *fne*^ts^** > +, 91.1 ± 1.0%; Figure 2B). In addition, knockdown of *fne* (*repo*^ts^** > *fne-IR*) did not affect thermal sensation. *Transient receptor potential cation channel A1* (*TrpA1*) is a nociceptor-related gene (Neely et al., 2011), and we used a *TrpA1* mutant (*TrpA1*^1^) as a positive control (avoidance rate was 17.3 ± 4.0%). Our data indicated that changes in *fne* expression in neurons and glia caused defects in thermal nociception, particularly in the flies with *fne* overexpression in glia.

### Impaired synaptic transmission of the larval neuromuscular junction in *fne*-knockdown flies

**FIGURE 3:**
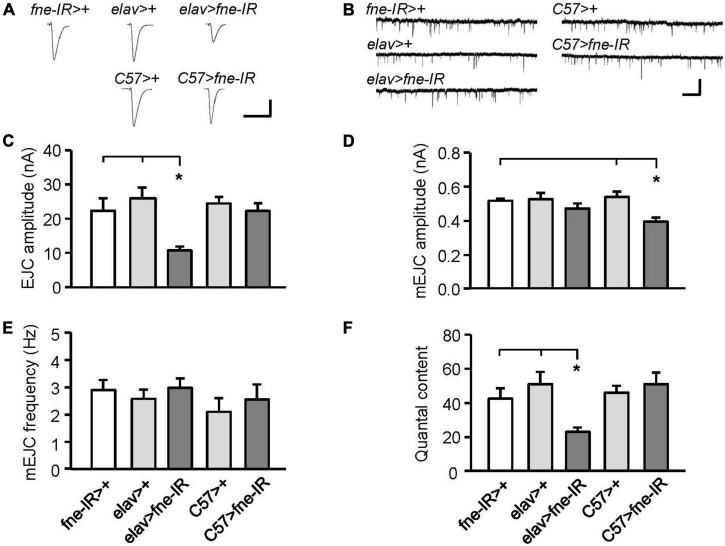
Knockdown of *fne* in neurons and muscle cells altered synaptic transmission in the larval NMJ. **(A)** Representative traces of EJC in the indicated genotypes. Calibration: 10 nA, 50 ms. **(B)** Representative traces of mEJC in the indicated genotypes. Calibration: 1 nA, 1 s. Quantification of **(C)** EJC amplitude, **(D)** mEJC amplitude, **(E)** mEJC frequency, and **(F)** quantal content. Error bars represent SEM. *p < 0.05, *n* = 8 for each group.

Changes in *fne* expression impaired phototransduction and adult behaviors, which might result from defective synaptic transmission. Thus, we performed an electrophysiological study of the third-instar larval NMJ for the effects of *fne* on synaptic transmission. Continuous recordings were made while the muscle fiber M12 was voltage clamped at –80 mV and segmental nerves innervating the muscle cell were stimulated by 0.05 Hz in a HL-3 solution containing 0.2 mM Ca^2+^ (Guo and Zhong, 2006). We examined an evoked EJC in the *fne*-knockdown flies (Figures 3A,C). The knockdown of *fne* in neurons (*elav* > *fne-IR*), which represents a presynaptic site of synaptic transmission, caused significantly smaller EJC amplitude (10.70 ± 1.16 nA) than the control flies (*elav* > + and *fne-IR* > +, 25.96 ± 3.13, and 22.31 ± 3.66 nA, respectively, *p* < 0.05). The knockdown of *fne* in muscle cells (*C57* > *fne-IR*), which represents a postsynaptic site of synaptic transmission, caused no difference in the EJC amplitudes among the control flies (*C57* > + and *fne-IR* > +). Because EJC amplitudes decreased only in the *elav* > *fne-IR* flies, it implies that their presynaptic function was affected.

Subsequently, we examined the amplitude and frequency of mEJCs to determine whether the spontaneous release of synaptic vesicles had changed (Figures 3B,D,E). The knockdown of *fne* in neurons did not alter the amplitude or frequency of the mEJCs. Only the mEJC amplitudes in the muscle cells expressing *fne*-*IR* (*C57* > *fne-IR*) significantly decreased compared to the control flies (*C57* > + and *fne-IR* > +, 0.40 ± 0.02 vs. 0.54 ± 0.03, and 0.52 ± 0.01 nA, respectively, *p* < 0.05). We also determined the quantal content, which reflects the number of vesicles released in response to a nerve impulse. A significant reduction in quantal content was observed in the *elav* > *fne-IR* flies (*elav* > *fne-IR*, 23.13 ± 2.50; *elav* > +, 50.99 ± 7.23; *fne-IR* > +, 42.53 ± 6.07, *p* < 0.05; Figure 3F).

### Impaired synaptic function of the larval neuromuscular junction in *fne*-overexpressed flies

**FIGURE 4:**
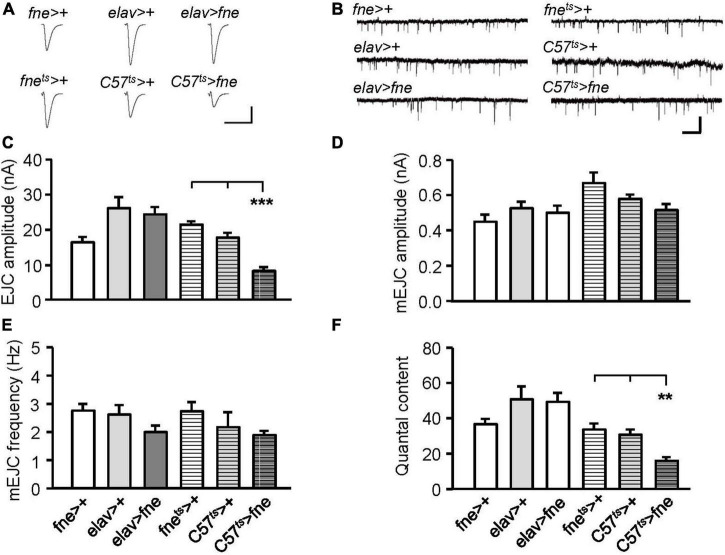
Overexpression of *fne* in muscle cells altered synaptic transmission in the larval NMJ. **(A)** Representative traces of EJC in the indicated genotypes. Calibration: 10 nA, 50 ms. **(B)** Representative traces of mEJC in the indicated genotypes. Calibration: 1 nA, 1 s. Quantification of **(C)** EJC amplitude, **(D)** mEJC amplitude, **(E)** mEJC frequency, and **(F)** quantal content. Bars with horizontal lines represented the flies that were reared at 18°C and shifted to 29°C for 1 day to induce *fne* expression. Error bars represent SEM. **p < 0.01, ***p < 0.001, *n* = 8 for each group.

We attempted to determine whether the overexpression of *fne* also affected synaptic transmission (Figures 4A,C). The overexpression of *fne* in muscle cells (*C57*^ts^** > *fne*) caused significantly smaller EJCs (8.20 ± 1.02 nA) than the control flies (*C57*^ts^** > + and *fne*^ts^** > +, 17.60 ± 1.39, and 21.36 ± 0.86 nA, respectively, *p* < 0.001). A significant reduction in quantal content was also observed in the *C57*^ts^** > *fne* flies (*C57*^ts^** > *fne*, 16.11 ± 1.99; *C57*^ts^** > +, 30.90 ± 2.91; *fne*^ts^** > +, 33.95 ± 3.30, *p* < 0.01; Figure 4F). The overexpression of *fne* in neurons (*elav* > *fne*) resulted in the EJC amplitudes and quantal content being similar with those of the control flies (*elav* > + and *fne* > +). In addition, the amplitude and frequency of mEJCs in the flies overexpressing *fne* in neurons and muscle cells did not differ from those of the controls (Figures 4B,D,E), suggesting that spontaneous synaptic transmission and constitutive synaptic vesicle fusion was not affected by *fne* overexpression.

### Knockdown of *fne* in the larval neuromuscular junction did not affect short-term synaptic plasticity

**FIGURE 5:**
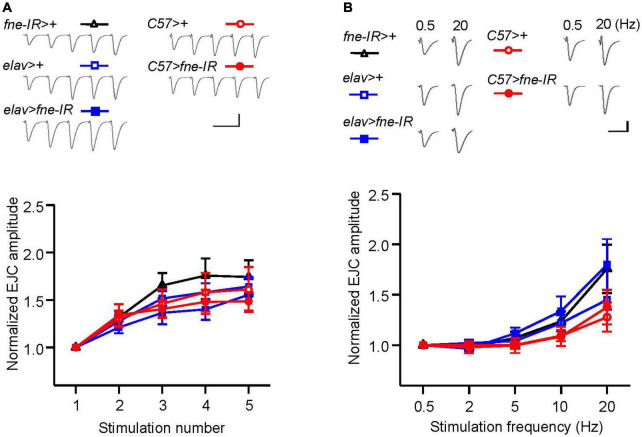
Knockdown of *fne* in neurons and muscle cells did not affect short-term synaptic facilitation in the larval NMJ. **(A)** STF during a short train of repetitive stimulation at 25 Hz in the wild type and in the knockdown of *fne* strains. Top, representative traces in the indicated genotypes. Bottom, summary of normalized EJC amplitude. **(B)** STF were determined at the various stimulation frequency of 0.5–20 Hz in the control and in the knockdown of *fne* strains. Top, representative traces of the EJCs for 0.5 and 20 Hz in the indicated genotypes. Bottom, summary of normalized EJC amplitude. Calibration: 10 nA, 50 ms. *N* = 8 for each group. Error bars represent SEM.

To determine whether *fne* is involved in short-term synaptic plasticity, we induced a short train of repetitive stimulation at 25 Hz after 5 min of baseline stimulation. The repetitive impulses affected the probability of neurotransmitter release and resulted in changes to synaptic efficacy over time, which reflected the electrical activity of the presynaptic terminals. Normal STF was observed in neurons and muscle cells expressing *fne-IR* (*elav* > *fne-IR* and *C57* > *fne-IR*; control groups: *elav* > *fne-IR* vs. *elav* > + and *fne-IR* > +; *C57* > *fne-IR* vs. *C57* > +, and *fne-IR* > +, respectively; Figure 5A). We also induced the frequency dependence of STF by delivering various frequencies (0.5–20 Hz). The synaptic transmission properties were normal with increasing stimulation frequency in the knockdown of *fne* in neurons and muscle cells (*elav* > *fne-IR* and *C57* > *fne-IR*; Figure 5B).

### Overexpression of *fne* in muscle cells affected short-term synaptic plasticity

**FIGURE 6:**
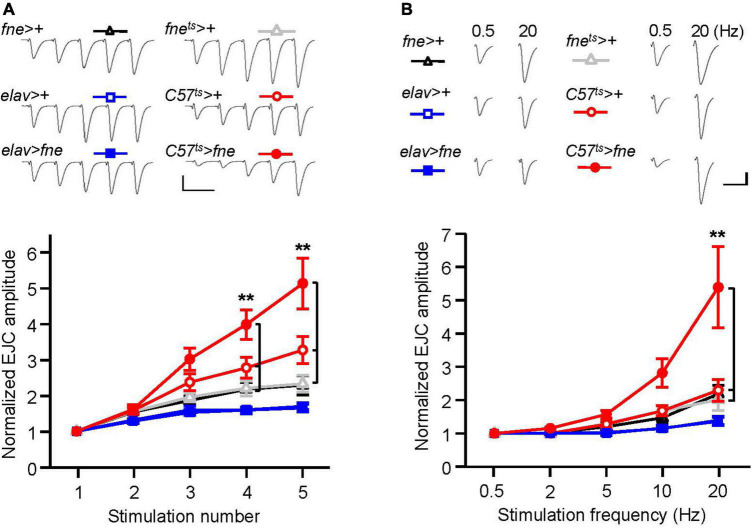
Overexpression of *fne* in muscle cells altered short-term synaptic facilitation in the larval NMJ. **(A)** STF during a short train of repetitive stimulation (25 Hz) in the wild type and in the overexpression of *fne* strains. Top, representative traces. Bottom, summary of normalized EJC amplitude. **(B)** STF were determined at the various stimulation frequency (0.5–20 Hz) in the control and in the overexpression of *fne* strains. Top, representative traces of the EJCs for 0.5 and 20 Hz in the indicated genotypes. Bottom, summary of normalized EJC amplitude. The *C57*^ts^** > *fne*, *C57*^ts^** > +, and *fne*^ts^** > + flies were reared at 18°C and shifted to 29°C for 1 day to induce *fne* expression. Calibration: 10 nA, 50 ms. *n* = 8 for each group. Error bars represent SEM. **p < 0.01.

To determine whether the overexpression of *fne* was involved in short-term synaptic plasticity, we induced a short train of repetitive stimulation at 25 Hz in the *fne*- overexpressing flies. Normal STF was observed in the neurons expressing *fne* (*elav* > *fne*), but facilitation increased significantly for *fne-*expressing muscle cells, by approximately 5.1-fold after repetitive stimulation, relative to the controls (*C57*^ts^** > *fne* vs. *C57*^ts^** > + and *fne*^ts^** > +; Figure 6A). A frequency-dependent facilitation was also obtained by stimulating muscle cells (*C57*^ts^** > *fne*), where the stimulation gradually increased in frequency from 0.5 to 20 Hz; however, no difference was observed in the neuronal *fne*-expressing flies (*elav* > *fne*; Figure 6B).

These results indicated that the knockdown of *fne* in neurons reduced the amplitude of EJCs and quantal content, whereas the overexpression of *fne* in muscle cells reduced the amplitude of EJCs and quantal content and increased STF, suggesting that *fne* plays an important role in synaptic transmission and plasticity.

### Knockdown of *fne* in glia affected synaptic transmission and short-term synaptic plasticity in the larval neuromuscular junction

**FIGURE 7:**
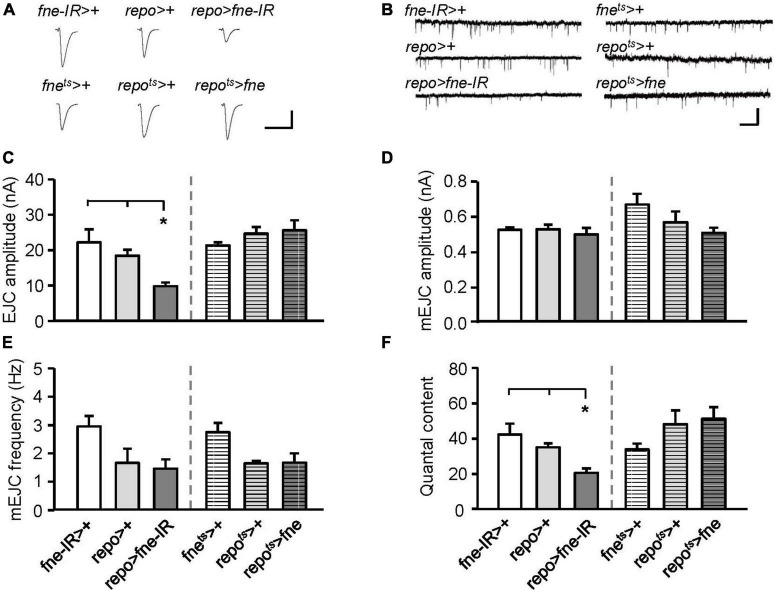
Change of *fne* expression in glia altered the synaptic transmission in the larval NMJ. **(A)** Representative traces of EJC while the glia expressing *fne* and *fne-IR*. Calibration: 10 nA, 50 ms. **(B)** Representative traces of mEJC in the indicated genotypes. Calibration: 1 nA, 1 s. Quantification of **(C)** EJC amplitude, **(D)** mEJC amplitude, **(E)** mEJC frequency, and **(F)** quantal content. Bars with horizontal lines represented the flies that were reared at 18°C and shifted to 29°C for 1 day to induce *fne* expression. Error bars represent SEM. *p < 0.05, *n* = 8 for each group.

Subsequently, we attempted to determine whether changes in *fne* expression in glia regulate synaptic transmission. The knockdown of *fne* in glia, rather than the overexpression of *fne* (*repo* > *fne-IR*), significantly reduced the amplitude of the EJCs in comparison with those of the EJCs of the controls (*repo* > + and *fne-IR* > +, 9.89 ± 1.03 vs. 18.53 ± 1.67 and 22.31 ± 3.66 nA, respectively, *p* < 0.05; Figures 7A,C). A significant reduction in quantal content was observed in the *repo* > *fne-IR* flies (*repo* > *fne-IR*, 20.83 ± 2.54 vs. *repo* > +, 35.35 ± 2.30; *fne-IR* > +, 42.53 ± 6.07, *p* < 0.05; Figure 7F). Changes in *fne* expression (*repo* > *fne-IR* and *repo*^ts^** > *fne*) in glia did not cause the amplitude or frequency of the mEJCs to differ from those of the controls (Figures 7B,D,E).

**FIGURE 8:**
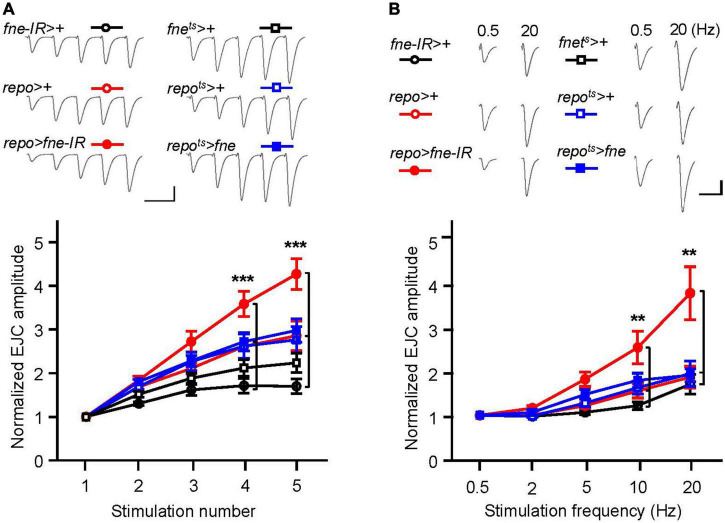
Change of *fne* expression in glia altered short-term synaptic facilitation in the larval NMJ. **(A)** STF during a short train of repetitive stimulation (25 Hz) in the control and in the glia expressing *fne* and *fne-IR*. Top, representative traces. Bottom, summary of normalized EJC amplitude. **(B)** STF were determined at the various stimulation frequency (0.5–20 Hz) in the control and in the glia expressing *fne* and *fne-IR*. Top, representative traces of the EJCs for 0.5 and 20 Hz in the indicated genotypes. Bottom, summary of normalized EJC amplitude. The *repo*^ts^** > *fne*, *repo*^ts^** > +, and *fne*^ts^** > + flies were reared at 18°C and shifted to 29°C for 1 day to induce *fne* expression. Calibration: 10 nA, 50 ms. *n* = 8 for each group. Error bars represent SEM. **p < 0.01, ***p < 0.001.

To determine whether changes in *fne* in glia are involved in short-term synaptic plasticity, we induced a short train of repetitive stimulation at 25 Hz. A significant increase in STF, approximately 4.5-fold, occurred after repetitive stimulation for *fne-IR* expression in glia (*repo* > *fne-IR*). However, a normal STF was observed in the *fne*-overexpressing flies (*repo*^ts^** > *fne*; Figure 8A). In addition, when STF was induced at 0.5–20 Hz, synaptic transmission revealed a frequency-dependent facilitation in the glial *fne-*knockdown flies (*repo* > *fne-IR*). This phenomenon was not observed in flies with *fne* overexpression in glia (*repo*^ts^** > *fne*; Figure 8B). The results indicated that the knockdown rather than overexpression of *fne* in glia reduced the amplitudes of EJCs and quantal content and increased STF, suggesting that *fne* in glia has an important role in synaptic transmission and plasticity.

### Changes of *fne* expression altered fly lifespan

**FIGURE 9:**
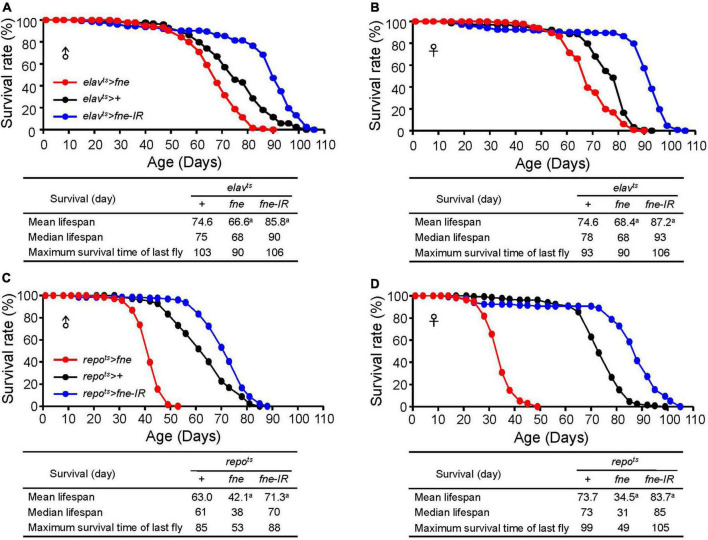
Effects of *fne* expression on the lifespan in neurons and glia. The survival curve of overexpression and knockdown of *fne* in neurons. Data represent the total lifespan of examined **(A)** male and **(B)** female flies. The genotypes of each group are *elav*^ts^** > *fne* (red circle), *elav*^ts^** > + (black circle), and *elav*^ts^** > *fne-IR* (blue circle). The total lifespan of overexpression and knockdown of *fne* in glia represents in **(C)** male and **(D)** female flies. The genotypes of each group are *repo*^ts^** > *fne* (red circle), *repo*^ts^** > + (black circle), and *repo*^ts^** > *fne-IR* (blue circle). Flies were reared at 18°C and transferred to 25°C after eclosion to induce *fne* and *fne-IR* expression. Total *n* = 120–130 for each group. Data are significantly different from the control at *^a^p* < 0.001.

To examine whether changes of *fne* expression can alter fly lifespan, we expressed *fne* and *fne-IR* in neurons and glia started at adult stage under *elav^ts^* and *repo^ts^* drivers, respectively, and measured their lifespan. Expression pattern of *elav-GAL4* and *repo-GAL4* lines was verified by crossing to a *UAS-GFP* (Supplementary Figure 2). As shown in Figures 9A,B, the mean lifespan decreased by 10.7 and 8.3% when overexpressing *fne* in neurons of male and female flies, respectively. Conversely, knockdown of *fne* expression in neurons increased up to 15.0 and 16.9% lifespan in both males and females, respectively. Similar results also observed when changing *fne* expression in glia (Figures 9C,D). The mean lifespan of *repo*^ts^** > *fne* flies significantly decreased by 33.2 and 53.2% in males and females, respectively, and in *repo*^ts^** > *fne-IR* flies, the mean lifespan was extended in both gender by 13.2% (males) and 13.6% (females). Our results suggest that changes of *fne* expression could alter fly lifespan. In particular, knockdown of *fne* expression in both neurons and glia started from the adult stage seems to appear a beneficial effect on longevity in both male and female flies.

## Discussion

The ELAV family of proteins have been determined to perform post-transcriptional processes and mediate important roles in the *Drosophila* nervous system. For example, neural-specific target transcripts, *erect wing*, *neuroglian*, and *armadillo*, which can interact with ELAV’s RRMs, are essential for the development and maintenance of neuronal function (Koushika et al., 2000). At least two target transcripts, *extramacrochaetae* and *bag-of-marbles*, are regulated by the RBP9 protein and regulate neuron and germ cell differentiation, respectively (Park et al., 1998; Kim-Ha et al., 1999). Few reports have identified the target transcripts of the FNE protein, and one target, *Basigin*, which encodes a cell adhesion molecule, accounts for the dendritic growth, and arborization in class IV da neurons during larval development (Alizzi et al., 2020). Little is known about the function of *fne* in neuronal plasticity. Recently, the knockdown of *fne* in muscle cells decreased the mEJC amplitudes. Changes in mEJCs can occur presynaptically (e.g., change in the number of neurotransmitters packed into a vesicle) or postsynaptically (e.g., change in the sensitivity of postsynaptic receptors). The overexpression of *fne* in muscle cells decreased the EJC amplitudes and quantal content and increased STF (all occurred presynaptically). Muscle-specific changes in *fne* expression affected neurotransmission, majorly on the presynaptic side. One possible mechanism is that FNE regulates the postsynaptic activity of calcium/calmodulin-dependent protein kinase II (*CaMKII*) through 3’UTR APA (Tiruchinapalli et al., 2008; Kuklin et al., 2017; Wei and Lai, 2022) and indirect alteration of CaMKII activity affects retrograde control of homeostasis of neurotransmitter release (Haghighi et al., 2003).

In addition, the loss of neuronal and glial *fne* resulted in presynaptic defects in neurotransmission, including smaller EJC amplitudes and quantal content, whereas normal transmission was observed in the overexpression of neuronal and glial *fne*. The loss of glial *fne* also increased STF (a presynaptic property), suggesting that *fne* is a critical regulator of neuronal transmission and synaptic plasticity. Several reports have characterized the role of glia in the PNS of *Drosophila*. The perineurial glial cells clear presynaptic debris through phagocytosis to modulate neuronal branching and shape synaptic connections during development or to do so in an activity-dependent manner (Fuentes-Medel et al., 2009). One purposed mechanism of glial *fne* in the regulation of synaptic transmission is through *genderblind* (*gb*). *fne* mutant affects 3’UTR APA of *gb* (Wei et al., 2020). Disruption of perineurial glial *gb* causes a reduction in extracellular free glutamate and results in increased postsynaptic glutamate receptor clustering and long-term strengthening of glutamatergic synapses at the NMJ (Sigrist et al., 2002; Augustin et al., 2007). Thus, glial cells also contribute to homeostatic plasticity, a process that modulates the presynaptic release of neurotransmitters, which is considered a compensatory response to the loss or inhibition of postsynaptic receptors (Wang et al., 2020). By converging the modulation of synaptic transmission with neurons, glial *fne* expression can influence neurotransmission between presynaptic and postsynaptic cells.

Although the ELAVL/Hu protein family has been determined to regulate neuronal development and differentiation, few reports have described their function in the retina. Amato et al. (2005) demonstrated the distribution of three members of the ELAVL/Hu family in *Xenopus* retinogenesis during retinal development, and Pacal and Bremner (2012) identified the expression patterns of Elavl2/3/4 in a developing murine retina. Elavl2 was also identified to express in retinal cells during retinogenesis and ablation of Elavl2 leads to deficit in ERG responses and visual acuity (Wu et al., 2021). The *elav* of *Drosophila* expresses in the imaginal disc of the larval eye and the adult optic lobe (Robinow and White, 1988). We found changes in the level of *fne* expression in both photoreceptors and glia beginning at the adult stage altered light-evoked synaptic potential. In the visual system of adult *Drosophila*, light stimuli depolarize photoreceptors to induce the release of histamines and activate postsynaptic receptors, histamine-gated Cl^–^ channels, on the lamina neurons (Chaturvedi et al., 2014). The ERG response can be modulated by the surrounding epithelial glia through the removal of histamines from the synaptic cleft (Stuart et al., 2007; Chaturvedi et al., 2014). Thus, for the retina, changes in *fne* expression in glia cause more severe defects than in neurons, particularly for the on- and off-transient amplitudes, suggesting a significant role of glial *fne* in phototransduction.

A study from Ustaoglu et al. (2021) found that elavl2 is required for learning and memory consolidation in honey bees. Another study showed that ELAVL2-regulated transcriptional and splicing networks in human neurons including RBFOX1 and FMRP-related pathway are involved in synaptic function and neural development (Berto et al., 2016). These results highlight ELAVL/Hu’s regulator of important neuronal pathways. For the lifespan experiment, our data showed that knockdown of *fne* in neurons and glia could cause the lifespan extension in both genders. However, genetic background effects might produce variable lifespan results, since we did not backcross at least 6 generations to produce the isogenic *UAS* strains (Piper and Partridge, 2016). Thus, we provided the tentative conclusion and further exploration need to be done for lifespan.

For the orthologs of *fne* in humans, FNE strongly shares its amino acid identity with ELAVL2/HuB and ELAVL4/HuD (Samson and Chalvet, 2003). Studies have indicated that ELAVL2/HuB and ELAVL4/HuD are highly enriched in the neuronal lineage but absent in glial cells (King et al., 1994; Mirisis and Carew, 2019; Hilgers, 2022). Defects in ELAVL2/HuB, ELAVL4/HuD, and their target transcripts can lead to abnormal neuronal proliferation and development and result in the pathogenesis of numerous CNS disorders, including seizures, autism, and schizophrenia (Yamada et al., 2011; Bronicki and Jasmin, 2013; Berto et al., 2016; Zybura-Broda et al., 2018). Silencing ELAVL1/HuR, which also shares high amino acid identity with FNE (Samson and Chalvet, 2003) in the activated microglia and astrocytes attenuate neuroinflammation and the occurrence of amyotrophic lateral sclerosis (Borgonetti et al., 2021). Thus, we conducted a bioinformatic analysis of *ELAVL2*/*HuB* and *ELAVL4*/*HuD* in neurons and glia from published gene expression datasets of human, mice, and rat samples. Two genes are expressed in all types of glia and neurons at similar levels (Supplementary Table 1). Similar human’s results also observed in the database of The Human Protein Atlas (Digre and Lindskog, 2021).

In the present, we found changes in the level of *fne* expression in neurons and glia affected phototransduction, adult behaviors, synaptic transmission of larval NMJ, and could influence the lifespan. Although the *Drosophila* ELAV protein can be detected in the majority of neurons, Berger et al. (2007) demonstrated that ELAV can transiently express in early embryonic glial cells but not in adults. The expression of *fne* in glia has never been reported. Our findings highlighted the important *fne*’s function not only in neurons but also in glia. As FNE is an RNA-binding protein, homeostatic maintenance of FNE stability and its downstream multiple pathways through target transcripts is crucial for brain activities. Further explorations into the physiological and molecular functions of glial *fne* are expected to clarify certain *Drosophila* behaviors, which may also be a feature of mammal brains.

## Data availability statement

The original contributions presented in this study are included in the article/Supplementary material, further inquiries can be directed to the corresponding author.

## Author contributions

W-YL and H-PL wrote the manuscript. C-HL, W-YL, and H-PL performed the experiments. JC carried out the bioinformatic analysis. H-PL supervised the research and funding acquisition. All authors have read and approved the final manuscript.
