# Supplementary material for: Alterations of RNA-binding protein found in neurons in Drosophila neurons and glia influence synaptic transmission and lifespan
Source: Front Mol Neurosci. 2022 Nov 11;15:1006455. doi: 10.3389/fnmol.2022.1006455 (PMC9693765; doi:10.3389/fnmol.2022.1006455)
Supplement: Supplementary file 1 [file Data_Sheet_1.PDF]

Supplementary Figure 1

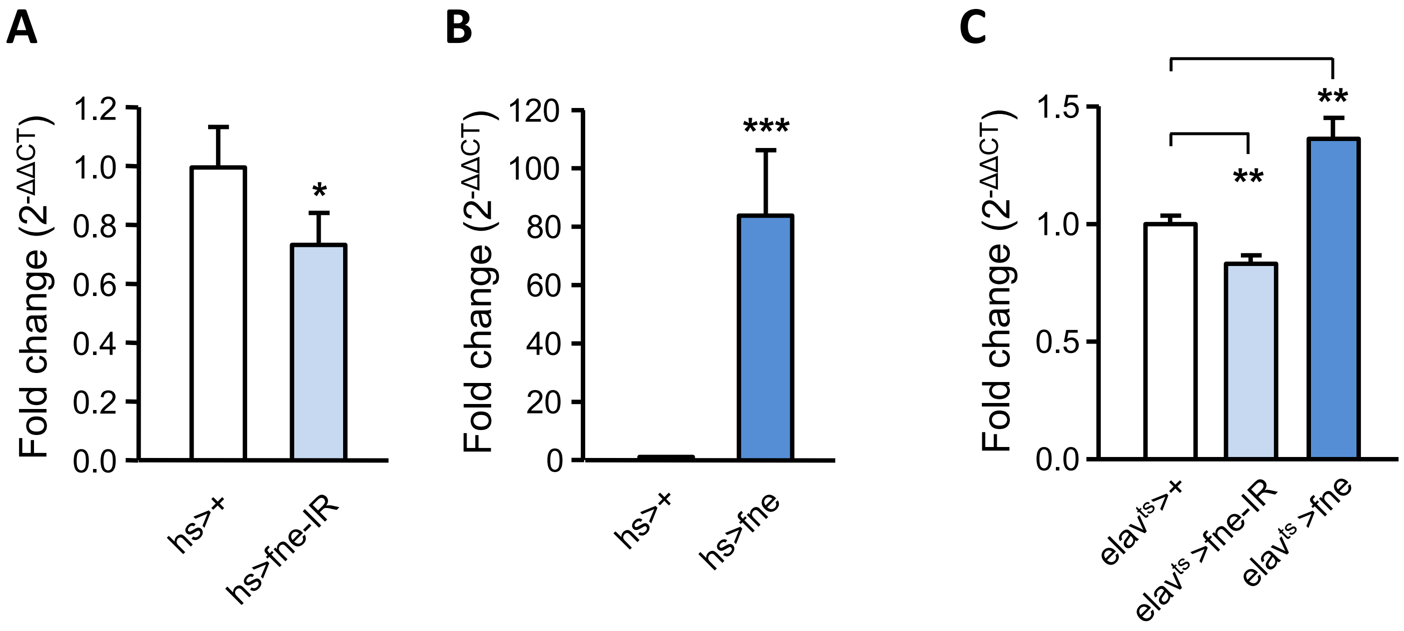

Supplementary Figure 1. Evaluation of *fne*-knockdown and *fne*-overexpression flies by qRT-PCR. (A) *hs>fne-IR* and (B) *hs>fne* flies were collected to evaluate *fne* transcript after 36 ˚C heat shock 1 h for five consecutive days. (C) *elav^{ts}>fne-IR*, and *elav^{ts}>fne* flies were reared at 18 ˚C and shifted to 29 ˚C after eclosion to evaluate *fne* transcript. Relative transcript abundances were normalized with *RpL32* as the internal standard. N = 3-4 and error bars represent SEM. Statical analysis were performed by a *Student*'s t-test. * $p < 0.05$, ** $p < 0.01$, *** $p < 0.001$. Primers sequences, *fne* F: 5'-catgacgcaggaggagatg-3'; *fne* R: 5'-gattgagagcggtcaacgat-3'; *RpL32* F: 5'-cggatcgatatgctaagctgt-3'; *RpL32* R: 5'-cgacgcactctgttgtcg-3'.

Supplementary Figure 2

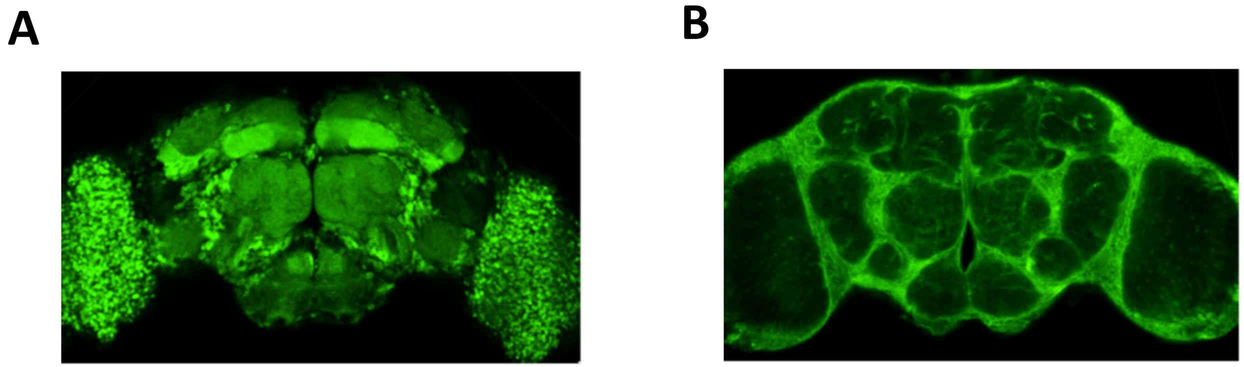

Supplementary Figure 2. Expression pattern of *elav*^{c155}*-GAL4* and *repo*^{7415}*-GAL4* was verified by GFP. (A) *elav-GAL4*^{c155}*>GFP and* (B) repo-*GAL4*^{7415}>GFP were showed GFP signals appeared in neurons and glial cells, respectively. (*UAS-GFP* strain: BDSC #32186)
