# Supplementary material for: Alterations of RNA-binding protein found in neurons in Drosophila neurons and glia influence synaptic transmission and lifespan
Source: Front Mol Neurosci. 2022 Nov 11;15:1006455. doi: 10.3389/fnmol.2022.1006455 (PMC9693765; doi:10.3389/fnmol.2022.1006455)
Supplement: Supplementary file 2 [file Table_1.DOCX]

**Supplementary Table 1. Summary of the sample size, NCBI GEO accession number, and expression levels of *ELAVL2/HuB* and *ELAVL4/HuD.***

| **Species** | **Anatomy** | **N** | **Sources** | **References**  **(PMID)** | ***ELAVL2***  (mean ± SD) | ***ELAVL4***  (mean ± SD) |
| --- | --- | --- | --- | --- | --- | --- |
| *Homo sapiens* | hippocampus pyramidal neuron | 28 | GSE5281  GSE9770 | 17077275  29937276 | 15.00 ± 1.70 | 13.74 ± 0.77 |
|  | astrocyte | 19 | GSE9834  GSE15824  GSE36634  GSE83670 | 19476540  21406405  19807941  27725112 | 11.86 ± 3.26 | 10.83 ± 3.13 |
|  | microglia cell | 5 | GSE29796  GSE36634 | 23727239  19807941 | 7.83 ± 0.50 | 10.79 ± 1.56 |
|  | oligodendrocyte progenitor cell | 27 | GSE29796 | 23727239 | 10.70 ± 1.98 | 11.27 ± 1.08 |
| *Mus musculus* | hippocampus neuron | 27 | GSE11679  GSE16496 | 18836535  21858037 | 13.69 ± 0.69 | 12.78 ± 0.53 |
|  | astrocyte | 158 | GSE6675  GSE29317  GSE35338  GSE9566  GSE13379  GSE69166  GSE18765  GSE66370  GSE5582  GSE69079  GSE69340  EBI/E-MTAB-5445 | (NA)  (NA)  22553043  18171944  20308160  26528138  21112568  26250529  17283119  28539349  26371510  28213522 | 10.39 ± 2.02 | 10.00 ± 1.74 |
|  | microglia cell | 17 | GSE10246  GSE29949  GSE55968 | 18442421  21788405  25690519 | 8.48 ± 0.65 | 9.72 ± 0.57 |
|  | oligodendrocyte progenitor cell | 14 | GSE9566  GSE30626 | 18171944  24453331 | 11.76 ± 0.9 | 13.19 ± 0.78 |
| *Rattus norvegicus* | hippocampus neuron | 48 | GSE12483  GSE13537  GSE13538 | 18973596  19109909  19109909 | 12.78 ± 0.87 | 12.29 ± 1.30 |
|  | astrocyte | 21 | GSE26066 | 21903074 | 11.54 ± 1.6 | 11.43 ± 1.69 |
|  | microglia cell | 6 | GSE29885 | 22697290 | 13.95 ± 0.8 | 11.69 ± 0.53 |
|  | oligodendrocyte progenitor cell | 8 | GSE11218 | 18987208 | 12.31 ± 1.22 | 11.76 ± 1.29 |
